# Oligomeric protein structure networks: insights into protein-protein interactions

**DOI:** 10.1186/1471-2105-6-296

**Published:** 2005-12-10

**Authors:** KV Brinda, Saraswathi Vishveshwara

**Affiliations:** 1 Molecular Biophysics Unit, Indian Institute of Science, Bangalore, India 560012

## Abstract

**Background:**

Protein-protein association is essential for a variety of cellular processes and hence a large number of investigations are being carried out to understand the principles of protein-protein interactions. In this study, oligomeric protein structures are viewed from a network perspective to obtain new insights into protein association. Structure graphs of proteins have been constructed from a non-redundant set of protein oligomer crystal structures by considering amino acid residues as nodes and the edges are based on the strength of the non-covalent interactions between the residues. The analysis of such networks has been carried out in terms of amino acid clusters and hubs (highly connected residues) with special emphasis to protein interfaces.

**Results:**

A variety of interactions such as hydrogen bond, salt bridges, aromatic and hydrophobic interactions, which occur at the interfaces are identified in a consolidated manner as amino acid clusters at the interface, from this study. Moreover, the characterization of the highly connected hub-forming residues at the interfaces and their comparison with the hubs from the non-interface regions and the non-hubs in the interface regions show that there is a predominance of charged interactions at the interfaces. Further, strong and weak interfaces are identified on the basis of the interaction strength between amino acid residues and the sizes of the interface clusters, which also show that many protein interfaces are stronger than their monomeric protein cores. The interface strengths evaluated based on the interface clusters and hubs also correlate well with experimentally determined dissociation constants for known complexes. Finally, the interface hubs identified using the present method correlate very well with experimentally determined hotspots in the interfaces of protein complexes obtained from the Alanine Scanning Energetics database (ASEdb). A few predictions of interface hot spots have also been made based on the results obtained from this analysis, which await experimental verification.

**Conclusion:**

The construction and analysis of oligomeric protein structure networks and their comparison with monomeric protein structure networks provide insights into protein association. Further, the interface hubs identified using the present method can be effective targets for interface de-stabilizing mutations. We believe this analysis will significantly enhance our knowledge of the principles behind protein association and also aid in protein design.

## Background

It is well known that a vast majority of cellular functions are mediated through protein-protein and protein-DNA interactions. Protein association is implicated in cellular signal transduction, antigen-antibody binding, in the regulation of gene expression and in the functioning of a huge variety of other constitutive multimers, where the multimeric state is the biologically active state. Hence, extensive research has been carried out to identify and to understand the underlying principles of protein association and interactions. Some insights to such interactions at atomic level have emerged from the analysis of large number of high-resolution crystal structures. Such investigations involve the characterization of the geometrical, chemical, and the energetic features of the interfaces as explained in the various reviews [1-6]. Specific studies include obtaining residue preferences at the interfaces [7], calculations of geometric parameters and shape complementarities between the interacting protein chains [8-11], calculations of the loss in accessible surface upon multimerization [12-15], elucidation of the role of hydrogen bonds, salt-bridges and hydrophobic and polar interactions at protein interfaces [16-21] and the analysis of conservation of residues at protein interfaces [22-26]. Various investigators have identified and analyzed energetic hot spots in protein interfaces using varied approaches [26-29]. Haliloglu *et al.*, have compared protein folding and protein binding using vibrational motions of interface hot spots and conserved residues and conclude that both processes involve similar packing of amino acid residues [30]. They also provide a method for identifying hot spots at binding interfaces. Further, Ofran and Rost have classified and analyzed the differences between six interface types including obligatory and transient homo and hetero oligomers [31]. De *et al.*, have also distinguished obligatory and non-obligatory interfaces using differences in the amino acid contacts and interactions patterns between the two interface types [32]. Bahadur *et al.*, have distinguished the biological oligomers from non-specific oligomers caused due to crystal packing [33]. There have also been speculations about whether folding and binding are completely de-coupled with each other or whether they occur simultaneously, one coupled with the other [34]. Wolynes and co-workers through simulations present that even if the monomers involved in binding may be stable separately, binding might preferably occur through unfolded intermediates, thus implying that folding and binding may be coupled in vivo and driven by the native state topology of the functional protein [34]. Further, a community-wide evaluation of the significance and success of different methods used in the prediction of protein-protein interactions and protein docking has been carried out (CAPRI) and has been hugely successful [35]. However, though there have been significant advances in methods of protein docking, those that are generally used in the identification of binding sites in monomer surfaces and the prediction of protein-protein interactions sites are far from satisfactory. Hence, newer approaches are required to get more insights into the factors contributing to protein-protein interactions.

We have earlier carried out an analysis on a limited set of twenty homodimers to understand the principles of protein-protein interactions from a graph perspective [36]. This analysis was directed towards identifying clusters of amino acid residues with strong interactions at the protein interfaces, the nature of the residues involved in these interface clusters and the accessibility and conservation of these interface cluster forming residues. We had also proposed a simple and straightforward method to identify interacting surfaces on protein monomers, which was highly successful in that dataset. The present study focuses on the network of amino acid interactions across protein interfaces and has been carried out on a larger dataset of protein homo as well as hetero multimers. Recently, Del Sol and co-workers have investigated protein-protein complexes from the small-world network perspective using parameters like clustering coefficients and betweenness, where the central residues identified at the interfaces, have been found to correlate with the experimentally determined hotspots [37]. Further, the same group also proposes the rewiring of the small-world networks at protein interfaces to form clusters of central residues at the interfaces [38]. The current analysis also considers the protein structure in its multimeric form as a network of non-covalently interacting amino acids. However, we use a different definition of nodes and edges than the ones used by Del Sol and co-workers [37,38], and have also incorporated an interaction strength term in the network construction and in the analysis of different parameters to understand the network topology of protein multimers. Since we know that protein-protein interactions are mainly mediated through non-covalent interactions, the connections (edges) between amino acids (nodes) are defined on the basis of the strength of the non-covalent interactions, as evaluated from the normalized number of contacts between them. The results are analyzed in terms of the network properties such as the hubs (nodes with greater number of edges) and clusters of amino acid residues in the protein complex at a given interaction strength, with particular focus at the protein-protein interface. Such an approach gives a global perspective of the interactions across the interface, which is difficult to obtain from pair-wise interaction or loss of accessible surface area analysis. For example, our earlier analysis on the clusters of interacting residues at the protein interface has given insights regarding the sequence signatures responsible for the different types of quaternary association in legume lectins[39] and has also helped in the identification of hot spots in the α-α dimeric interface of *Escherichia coli *RNA polymerase[40]. The network representation presented here has also been used earlier to identify structural domains and domain interface residues in multi-domain protein using a graph spectral method [41]. However, in this analysis, we focus on the identification and analysis of amino acid clusters and hubs at protein-protein interfaces based on a generic network approach.

Interesting observations made from the present analysis on protein multimers include the fact that the strength of interfaces evaluated using the interface clusters and hubs identified by present method correlate well with the kinetic and thermodynamic parameters of complex formation evaluated experimentally. Further, the interface hubs identified here also correlate well with the experimentally identified hot spots on the basis of binding free energy. This result indicates that hotspots can be associated with interface hubs, the identification of which can be useful in rationally designing interface de-stabilizing mutants. Further, a comparison of the interface hubs to the hubs within the protein monomer and with the non-hubs at the interface show significant differences in the interface hub properties, such as the contribution of the charged interactions being considerably higher at the interfaces. The analysis of the interface clusters has also shown that the protein interfaces are as strong as or stronger than the protein cores in more than half the protein complexes considered in the dataset. Thus, the present algorithm has given a new perspective into analyzing protein structures in general and protein complexes in specific, which has shed light onto some of the factors involved in protein association.

## Results and discussion

The concept of networks in biology has been explored in the areas of protein interaction networks, metabolic networks etc [42]. The idea of considering protein structures as a network of amino acid connections is relatively new and has provided insights into protein structure, stability and folding. For instance, Vendruscolo *et al.*, and Dokholyan *et al.*, [43-45] have used a similar approach to understand protein folding, where as Atilgan *et al.*, [46] and Green and Higman [47] have represented protein structures as amino acid networks to analyze residue fluctuations and stability of the protein structures. Del Sol and O'meara have analyzed protein complexes as small-world networks where the central residues in the interfaces correlate with experimental hot spots [37,38]. We have previously used a similar network representation to understand the factors affecting protein stability where the amino acid residues are the nodes in the protein structure network and the strength of the non-covalent interactions between them are evaluated for the edge-determining criterion [48]. In the present work, this approach has been extended to protein quaternary structures rather than just protein tertiary structures so as to understand the factors responsible for protein association. We have extracted the interface cluster (a set of connected residues) and hub (a highly connected residue) information from the network representation of protein multimers as explained in the methods section. This has given insights into the role of specific amino acid residues in stabilizing inter-subunit interfaces. The hubs in many real-world networks are known to provide robustness to the networks against random attack[42]. However, targeted attacks on these hubs are known to destabilize them. In the multimeric protein structure networks, the interface hubs can be considered as the centers providing stability to these networks due to their extensive interactions and their presence at the oligomeric interface. Hence, the mutation of a hub can lead to the destabilization of the interface. Therefore, the hubs can be identified as hot spots at protein interfaces that can be targeted for interface de-stabilizing mutations.

A non-redundant set of 455 protein oligomers is used in this study. The oligomeric protein structures as a whole are represented as graphs, with each amino acid as a node and the strength of non-covalent interactions (I, evaluated as given in the methods section) between them determining the edges. Those amino acid pairs with interaction strength greater than a user-defined cutoff (I_min_) are connected by edges. Such graphs generated at various I_min _values, have been analyzed in this section to understand the details of protein-protein interfaces at the network level. Specifically, (1) the analysis of the interface clusters (defined as distinct clusters of amino acid residues with contributions from more than one chain of the protein oligomer) and interface hubs (defined as amino acid residues interacting with five or more residues with at least one residue belonging to a different chain than itself) have been presented. (2) The strength of interface interaction, as measured from the clusters and hubs identified at different I_min _values has been compared with the experimentally determined dissociation constants for known complexes. Finally, (3) the relevance of interface hubs to the stability of the oligomer is pointed out comparing some of the identified interface hubs with experimental results.

### Analysis of interface clusters

#### Correlation of interface clusters with loss of accessible surface area and composition of interface clusters

Interface clusters have been identified and analyzed for the loss of accessible surface area, the interface cluster composition and strength of the interface clusters based on I_min _and number of residues participating in interface cluster composition. The results of these investigations have been summarized in the two figures in the additional material (Additional file 1, Figures A1 and A2). The comparison of the residues that formed interface clusters at I_min _= 6% with those that have lost accessible surface area on oligomerization (δASA) showed a very good correlation (correlation coefficient = 0.83, Figure A1) indicating that the clusters identified at I_min _= 6% are a good representation of the oligomeric interfaces. Hence, all generic cluster analyses are carried out at this I_min_. This correlation decreases with increase or decrease of I_min _since higher I_min_s give specific strong clusters that fail to represent the complete interface and at lower I_min_, the monomeric protein core also becomes a part of the interface cluster. The interface cluster composition at I_min _= 6% also correlated very well with the residue composition obtained from δASA calculations (Figure A2) with preference for residues like Arginine, Histidine, Tryptophan, Tyrosine and Phenyl Alanine, though other residues are not left out. Such preferences have also been observed in several earlier interface analyses [7,15,33,36]. The present investigation in addition has provided information regarding the size and strength of oligomeric protein interfaces, through the parameters such as the number of interface clusters, the number of residues constituting the interface clusters and the size of the largest interface cluster. This is discussed in detail in a later section where experimental dissociation constants are compared with the amino acid cluster and hub results from our analysis.

#### Largest cluster analysis

**Figure 1:**
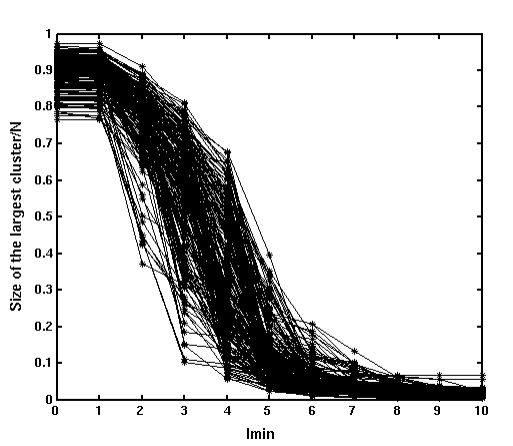
Size of the largest cluster/N (N = No. of amino acid residues in the protein structure) Vs I_min _for a set of oligomers from the dataset.

The size of the largest cluster is one of the parameters that are generally used to analyze the behavior and properties of complex networks [42]. Here we have identified the largest cluster and its size (in terms of number of residues) in the protein complexes considered in the present dataset at varying I_min _values. A plot of normalized size of the largest cluster (normalized with respect to the total number of residues in the complex) Vs I_min _is shown in Figure 1. Interestingly, all the protein multimers show a very similar profile of the largest cluster plot (Figure 1) with a transition around I_min _= 4%. (Incidentally, such a profile was also observed in the case of monomers, with transition around the same I_min _value [48]). The largest cluster in the complex at a given I_min _however, may or may not include the interface region. Undoubtedly, at I_min _= 0% the largest cluster includes the interface in all the cases, since the whole protein exists as one big cluster at this I_min_. As the I_min _is increased, the cluster size decreases and the largest cluster may be within the monomer or at the interface. This feature depends on the specific nature of the multimer complex and can be used to evaluate the strength of the interface with respect to the core of monomers. Since the clusters obtained at I_min _= 6% are significantly strong, this I_min _can be used to identify the strength of the interface using the following criterion. If the largest cluster at this I_min _is found at the interface, then it is a strong interface, where the interface is stronger than its monomeric core. Our analysis has shown that 291 protein multimers in a dataset of 455 proteins have such strong interfaces. The PDB list of these 291 proteins is given in the Additional file 1, Table A1a. The rest of the dataset that do not form such strong interfaces is given in the Additional file 1, Table A1b.

#### Identification of interface patches

**Figure 2:**
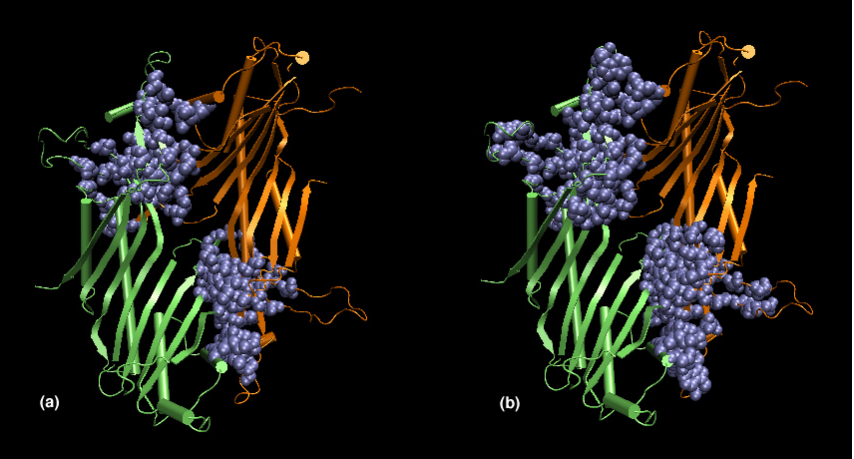
Interface amino acid clusters in Urate oxidase (1UOX) at (a) I_min _= 6% and (b) I_min _= 4%. The protein monomers and the cluster forming residues are colored differently and are shown in cartoon and van der Waal's representations respectively. This is an example of an interface with more than one patch at higher I_min_s that do not merge at lower I_min_s (1UOX) as identified from the interface clusters at different I_min_s.

Apart from the interface strengths, the present study involving interface cluster analysis also enables us to identify the number of interacting regions or patches that constitute the interface. For example, if a protein dimer shows two interface clusters at a higher I_min _(6%), which do not merge at a lower I_min _(4%), then it clearly indicates the presence of more than one patch in the interface. This is shown in Figures 2a and 2b, which show the interface clusters obtained in the protein dimer of Urate oxidase at I_min _= 6% and I_min _= 4% respectively. The dimer forms two distinct interface clusters that are strong and independent at both I_min _= 6% and at I_min _= 4% without merging at the lower I_min_. This indicates the presence of two separate patches at the interface. Thus, the present method of interface cluster analysis can provide information regarding the size, strength and the constitution of interfaces involved in protein oligomers.

### Analysis of interface hubs

#### Hub composition at interfaces

**Figure 3:**
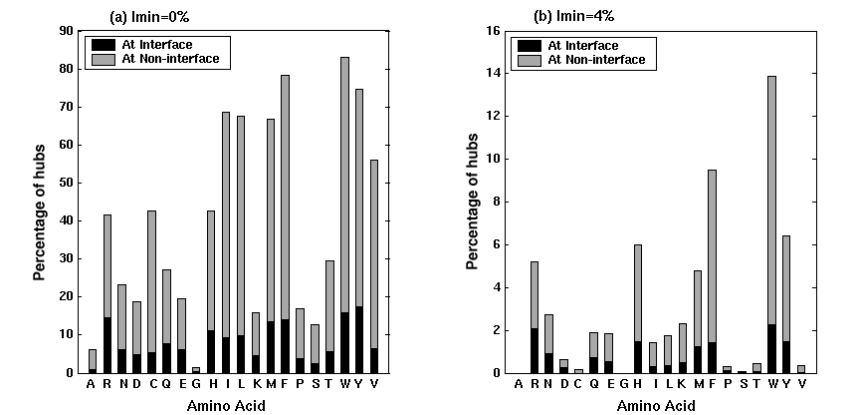
Amino acid preferences in the interface and the non-interface hubs at (a) I_min _= 0% and (b) I_min _= 4%. The percentage compositions of amino acid hubs in the interfaces and non-interfaces are presented (i.e., (No. of interface or non-interface amino acid hubs of type 'i' ÷ Total No. of amino acid residues of type 'i' present in the dataset) × 100).

The interface hubs are defined as those residues, which interact with five or more residues, out of which at least one residue belongs to the other monomer. Unlike interface cluster analyses (carried out mainly at I_min _= 6%), analyses of interface hubs are carried out from I_min _= 0% to I_min _= 4%, because, beyond I_min _= 4%, we do not get significant number of interface hubs for statistically significant results. The residue composition of the interface hubs, identified at I_min _= 0% and I_min _= 4% are presented in Figure 3. The hub composition of the non-interface regions (i.e., the other regions of the protein multimer devoid of the interface) at these I_min_s is also presented in the figure so as to compare the residue composition of the hubs in the interfaces and the non-interface regions. The values presented in the figure are percentage compositions with respect to the residue composition in the complete dataset.

It is evident from Figure 3 that Arginine, Tryptophan, Tyrosine, Phenyl Alanine, Histidine and Methionine are highly preferred as hubs at the protein interfaces at both higher and lower I_min_s, making them the strong interface hubs. The interface hub preferences of hydrophobic Leucine, Isoleucine and Valine are seen at lower I_min_s, making them the weak interface hubs. This overall profile is similar to the non-interface hub preference profile (Figure 3, [48]). However, there are some differences between the interface and non-interface hub preferences as can be seen from Figure 3. These include the fact that the interface hub preferences for the hydrophobic and aromatic residues are much lower when compared to that in the non-interface regions at the same I_min_. Further, the interface hub preferences of the charged residues are comparable to their non-interface hub preferences, though the non-interface regions are much larger than the interface regions. The differences between the preferences in the interface and the non-interface hubs become pronounced at higher I_min_s, with the predominance of Arginine and other charged amino acids in the interface hubs where as the aromatic residues predominate the non-interface hubs at higher I_min_s. The percentage of charged hubs is much higher in the interface regions than the non-interface regions and the percentage of aromatic and hydrophobic hubs is higher in the non-interface regions than the interface regions. Further, Arginine seems to make more contribution at the interface at both high and low I_min_s, ahead of the aromatic and hydrophobic amino acids (except a slight preference for Tyrosine and Tryptophan over Arginine at I_min _= 0%), unlike the non-interface hubs, where either the hydrophobic or aromatic residues or both are preferred ahead of Arginine at any I_min_. This shows that the protein interfaces have major contributions from the charged amino acid residues. The preference of Arginine and charged interactions at the protein interfaces has also been shown by a few previous analyses [7,15,17,33,36]. The present analysis also confirms this aspect with the charged interactions dominating the interfaces to a large extent.

Another important observation that can be made by comparing Figure A2 (see Additional file 1) and Figure 3 is that although the residues like Leucine, Isoleucine, Valine and Lysine are found significantly in the interface clusters even at higher I_min_s, they are not preferred as interface hubs at these I_min_s. Hence, there is marked difference in the residue preferences in interface clusters and the hub preferences at the interfaces.

#### Preferences of hub-forming residues to interact with other residue types

We have already seen the differences between the hub preferences in the interfaces and the non-interface regions from Figure 3. It would also be interesting to identify the preferences in the amino acid interactions that can lead to formation of the strong hubs at protein interfaces. Although the interacting preferences at the protein interfaces have been studied earlier [7], we present the same from the hub perspective here. The 20 × 20 matrix giving the preference of each of the 20 amino acid hubs to interact with themselves and with the other 19 residues at the interface regions (normalized percentages) at I_min _= 4% are presented in Table 1. The percentage of hubs of a particular residue type is also given. The obvious interaction preferences are between the positively charged Arginine and the negatively charged Aspartate and Glutamate, the interactions of the aromatic residues with other aromatic and hydrophobic residues and the preferences of the hydrophobic residues for other hydrophobic and aromatic residues. However, there seem to be some interesting preferences, apart from the normally seen salt-bridge, hydrogen bonds and aromatic stacking interactions. The significant ones include the preference of Arginine hubs to interact with itself in spite of its positive charge. Similarly, the preference of Histidine hubs to interact with Asparagine, Proline hubs with Phenyl alanine and Tyrosine, Leucine hubs with Arginine and Tyrosine hubs with Arginine and other charged residues are also noticed. There seems to be a preference for charged and polar interactions and those involving planar charge de-localized systems. A few examples are presented in detail in the next section.

**Table 1:** Preferences of interface hubs to interact with other residues at I_min _= 4%^1^

**Res**	**H_i_**	**Ala**	**Arg**	**Asn**	**Asp**	**Cys**	**Gln**	**Glu**	**Gly**	**His**	**Ile**	**Leu**	**Lys**	**Met**	**Phe**	**Pro**	**Ser**	**Thr**	**Trp**	**Tyr**	**Val**
ALA	0.00	0.00	0.00	0.00	0.00	0.00	0.00	0.00	0.00	0.00	0.00	0.00	0.00	0.00	0.00	0.00	0.00	0.00	0.00	0.00	0.00
ARG	2.06	1.56	10.08	5.74	14.32	0.45	4.62	15.15	0.50	3.34	3.90	3.96	2.28	2.34	6.24	2.79	5.40	4.51	2.45	6.63	3.73
ASN	0.92	3.70	4.27	10.67	9.96	0.43	8.11	7.54	2.56	5.97	2.42	4.27	2.13	2.42	4.84	3.70	2.84	6.40	2.42	6.26	9.10
ASP	0.27	1.51	18.87	3.02	4.15	0.00	3.02	5.28	0.00	7.92	0.38	5.66	2.64	1.89	5.28	6.79	7.55	9.43	5.66	9.06	1.89
CYS	0.00	0.00	0.00	0.00	0.00	0.00	0.00	0.00	0.00	0.00	0.00	0.00	0.00	0.00	0.00	0.00	0.00	0.00	0.00	0.00	0.00
GLN	0.70	1.35	5.84	8.09	5.62	0.90	12.58	7.64	0.45	2.47	3.82	8.31	1.35	4.04	6.52	1.80	3.82	6.74	3.60	9.66	5.39
GLU	0.53	1.04	20.42	3.11	2.60	1.21	3.46	3.29	0.87	7.44	6.23	4.84	6.06	3.29	7.44	2.60	6.06	7.61	2.60	7.79	2.08
GLY	0.00	0.00	0.00	0.00	0.00	0.00	0.00	0.00	0.00	0.00	0.00	0.00	0.00	0.00	0.00	0.00	0.00	0.00	0.00	0.00	0.00
HIS	1.48	1.18	7.24	12.46	7.24	1.52	3.03	5.89	0.84	12.12	4.04	5.72	2.53	4.71	2.69	0.34	5.72	6.90	3.54	5.56	6.73
ILE	0.31	0.33	6.31	1.66	3.32	0.00	8.64	6.31	0.33	3.32	6.64	9.63	3.32	5.32	14.95	2.33	5.65	4.65	4.65	6.64	5.98
LEU	0.33	2.56	11.44	3.75	5.92	0.79	6.51	4.14	1.38	5.72	6.31	8.09	1.78	1.18	7.69	4.54	3.94	2.37	4.34	6.31	11.24
LYS	0.48	2.52	5.03	5.49	13.04	1.14	4.35	17.39	1.14	3.89	3.43	5.49	0.23	3.66	3.20	1.37	8.47	5.72	2.75	9.15	2.52
MET	1.22	4.99	6.07	7.81	5.64	1.95	3.69	3.25	1.30	3.90	5.21	8.46	3.90	7.81	8.68	2.82	5.42	1.74	3.25	7.59	6.51
PHE	1.40	1.23	5.53	3.89	5.53	1.13	4.10	2.97	1.02	3.89	8.09	7.89	2.15	4.61	14.45	2.97	3.48	4.51	3.69	10.86	7.99
PRO	0.11	0.00	5.81	5.81	6.98	1.16	0.00	3.49	0.00	4.65	0.00	4.65	0.00	0.00	10.47	13.95	6.98	3.49	5.81	20.93	5.81
SER	0.06	0.00	9.09	0.00	9.09	0.00	16.36	0.00	0.00	7.27	0.00	0.00	0.00	0.00	10.91	0.00	16.36	23.64	7.27	0.00	0.00
THR	0.05	0.00	0.00	14.00	4.00	0.00	26.00	12.00	0.00	6.00	6.00	0.00	8.00	0.00	12.00	2.00	2.00	6.00	0.00	0.00	2.00
TRP	2.28	1.95	5.07	8.38	4.29	0.39	7.21	3.51	0.97	3.51	7.80	7.21	3.90	2.53	11.31	2.73	2.92	7.02	3.31	11.31	4.68
TYR	1.47	0.56	11.26	6.31	7.77	0.56	5.07	7.21	1.69	4.17	5.07	5.52	5.29	2.48	7.09	6.64	2.93	2.48	3.60	8.45	5.86
VAL	0.03	0.00	5.56	0.00	2.78	0.00	5.56	5.56	0.00	5.56	0.00	11.11	0.00	0.00	36.11	0.00	0.00	0.00	8.33	16.67	2.78

^1^1: The rows of the table (i) correspond to the twenty different types of amino acids forming the interface hubs. The second element in each row gives H_i_, the percentage of amino acids of each type 'i' forming interface hubs in the dataset (with respect to the total number of residues of type 'i' in the dataset). Elements three to twenty two in each row correspond to the percentage of interactions that the amino acid hub of type 'i' makes with the twenty different amino acid types (j) [with respect to the total number of interactions made by hub residue type 'i']. This gives the 20 × 20 matrix for the interactions of the interface hubs with other residues at I_min _= 4%. Thus, each ij^th ^element in the 20 × 20 matrix gives the percentage interactions of interface hub type 'i' with residue type 'j'.

It is to be noted that a similar 20 × 20 matrix for the non-interface hubs at I_min _= 4%, shows a different profile (see Additional file 1, Table A2), where the Arg-Arg, His-Asn, Leu-Arg, Tyr-charged and Tyr-polar interaction preferences are much lower than what is observed for the interface hubs shown in Table 1. In the non-interface hubs, the Tyr-Aromatic and Tyr-Hydrophobic interactions are more preferred than Tyr-charged or Tyr-polar interactions. Similarly, Arg-Aromatic interactions are also more preferred than Arg-Arg interactions and Leu-Leu and Leu-Phe are more preferred than Leu-Arg in case of the non-interface hubs.

#### Interactions of interface hubs

We have seen from Figure 2 and Table 1 that Arginine, Histidine and Tyrosine form some of the important hubs in the protein interfaces with some interesting interacting partners. We will discuss the interactions of some of these interface hubs in this section.

##### (a) Arginine hubs

**Figure 4:**
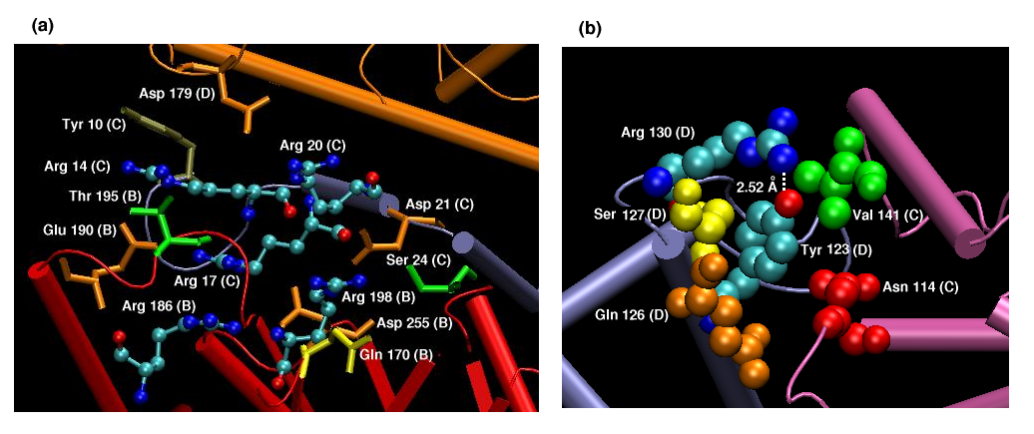
Examples of Arginine and Tyrosine hubs and their interactions. (a) Arginine hubs in the interface of 5-aminolevulinic acid dehydratase tetramer (1B4K) at I_min _= 4%. The protein tetramer is shown in cartoon representation with each monomer colored differently. Arg 17 (C), Arg 14 (C) and Arg 198 (B) form hubs, which interact with other residues (including other Arginines) belonging to different chains, thus forming a connected network of amino acid cluster at the interface. All the Arginine residues are shown in ball and stick representation and are colored according to the atom types (carbon in cyan, oxygen in red and nitrogen in blue) and the other residues are shown in bond representations and are colored according to the different residue types. The residue names and numbers are indicated along with the chains to which they belong. (b) Tyrosine hub in the interface of the shaker potassium channel (1A68) obtained at I_min _= 4%. The protein backbone is shown in cartoon representation with the monomers colored differently. The interface hub residue (Tyr 123 D) and the residues with which it interacts (Arg 130 D, Ser 127 D, Gln 126 D, Val 141 C, Asn 114 C) are shown in van der Waal's representation. The residue numbers, names and the chain identifiers are given. The Tyrosine hydroxyl is involved in a short-strong hydrogen bond with the Arginine side-chain with a donor-acceptor distance of 2.52 Å. The Tyrosine and the Arginine residues involved in the short hydrogen bond are colored according to atom type (carbon in cyan, nitrogen in blue and oxygen in red) and the other residues are colored differently based on their residue types.

Arginine has been shown to play a major role at protein interfaces [15,17,33,36]. In the present analysis, we find that there is a preference for Arginine in the interface clusters and in the interface hubs in comparison to the other amino acid residues. We also find that the interface Arginine hubs interact significantly with other Arginine and aromatic residues from the same chain and from other chains, apart from the normal salt-bridge interactions that they are most commonly involved in. Figure 4a shows some of the details of the interactions made by the interface Arginine hubs, which form a large interface cluster in 5-aminolevulinic acid dehydratase tetramer. Here, there are three Arginine hubs (Arg 17 C, Arg 14 C and Arg 186 B) and one of the Arginine hubs (Arg 17 C) interacts with four other Arginine residues (Arg 14 C, Arg 20 C, Arg 186 B and Arg 198 B), coming from two different chains, simultaneously. Moreover, the Arginine residues are also found to form stacking interactions with the π system of the aromatic Tyrosine residue and hydrogen bonds with Threonine, Serine and Glutamine side-chains. Further, there are negatively charged Asparate, Glutamate and Glutamine residues generously spread over this Arginine cluster, which neutralize the positive charges coming from the four Arginine residues. Arg-Arg stacking can also be seen along with hydrogen bonds involving the backbone oxygen of Arginine with backbone or side chain nitrogens of other Arginines. Investigations carried out on many other interface Arginine hubs showed that the Arg-Arg interactions can occur through a variety of interactions including planar stacking of the guanidine groups, hydrogen bonding between the guanidine-guanidine groups or guanidine group with main chain atoms, CHO hydrogen bonding of backbone oxygen with the Cβ, Cγ and Cδ of the Arginine side chain. One of the notable factors is that the Arginine hubs are invariably neutralized by the presence of negatively charged Glutamate and Aspartate side chains in and around the hub (need not necessarily form direct salt bridges), which have an overall neutralizing effect on the local environment. Thus, the versatile Arginine side-chain has been found to make extensive interactions stabilizing the oligomeric protein interfaces.

##### (b) Tyrosine hubs

One of the significant contributions to the interface hubs comes from the Tyrosine hubs, which makes extensive interactions with the charged and polar residues like Arginine, Aspartate, Asparagine, Glutamate and Glutamine apart from the expected interactions with the other aromatic residues and itself as can be seen from Table 1. The interactions of Tyrosine with charged and polar residues are generally due to hydrogen bonding or cation-π interactions. Figure 4b shows an example of an interface Tyrosine hub (Tyr 275) making different kinds of interactions including a short hydrogen bond involving the hydroxyl group (with Arg 282, donor-acceptor distance = 2.52 Å) at I_min _= 4%. (Tyrosine is also known to contribute to the stability of protein tertiary structure by means of short hydrogen bonds [49]). This Tyrosine residue also interacts with a Serine (279), Valine (141), Glutamine (278) and Asparagine (114) with Asparagine and Valine being from the other chain. Thus, we find that the Tyrosine residue is also versatile in its interactions due to its planar de-localized side chain and the hydroxyl group.

#### Statistics of hub versus non-hub interactions at the interface

The pair-wise residue interactions across the interface can be categorized into three with respect to the hub status of the interacting residues: (a) Hub-Hub (b) Hub-Nonhub and (c) Nonhub-Nonhub interactions. The percentage of the charged and hydrophobic interactions in these categories at I_min _= 0% and I_min _= 4% are given in Table 2. It can be seen from the table that the charged interactions dominate the hub-hub, hub-nonhub and nonhub-nonhub interactions at I_min _= 4% with a very high percentage in the hub-hub interactions. However at I_min _= 0%, the charged interactions still dominate the nonhub-nonhub interactions, where as the hydrophobic interactions dominate the hub-hub and hub-nonhub interactions at the same I_min _with a very high percentage in the hub-hub interactions. Therefore, when the I_min _is varied, the profile changes dramatically for the interactions involving the hubs (hub-hub and hub-nonhub), whereas there is no change in the overall profile in the nonhub-nonhub interactions. It is evident from Table 2 that the charged and hydrophobic interactions undergo a clear role reversal as far as hub-hub interactions at I_min _= 0% and 4% are concerned. This is also consistent with the residue preferences in the interface hubs shown in Figure 2, where the preferences change from Aromatic/hydrophobic to charged/Aromatic when I_min _is increased from 0% to 4%. Further, as we move from nonhub-nonhub to hub-hub interactions at I_min _= 4%, the charged+polar interactions including salt bridges as well as the aromatic-aromatic interactions increase, where as the hydrophobic interactions decrease. However the same at I_min _= 0% shows an increase in hydrophobic and aromatic interactions and a decrease in charged+polar interactions and salt bridges. In all cases, the hub-nonhub interactions fall in the intermediate category between the hub-hub and nonhub-nonhub interactions. These statistics clearly show a distinct profile for the interactions involving the hub residues when compared to those of the non-hub residues at the oligomeric protein interfaces.

**Table 2:** Statistics of interface interactions

**Interaction Type^6^**	**Nonhub-Nonhub**	**Hub-Nonhub**	**Hub-Hub**
I_min _= 4%			
Charged+Polar interactions^1 ^(Salt bridges)^3,5^	34% (11%)	37% (12%)	44% (13.7%)
Hydrophobic interactions^2 ^(Aromatic-Aromatic interactions)^4,5^	26.5% (3.2%)	24% (5.7%)	22% (9%)

I_min _= 0%			
Charged+Polar interactions (Salt bridges)	35.3% (8%)	24.3% (7%)	21.5% (6%)
Hydrophobic interactions (Aromatic-Aromatic interactions)	23% (0.4%)	32% (1%)	40% (5%)

^1^Charged and polar interactions involve Arg, Lys, His, Glu, Asp, Gln, Asn, Ser, Thr, Cys.

^2^Hydrophobic and Aromatic interactions involve Leu, Ile, Val, Ala, Gly, Met, Pro, Phe, Tyr and Trp.

^3^Salt bridges are between Arg/His/Lys and Glu/Asp.

^4^Aromatic interactions involve Phe/Tyr/Trp with themselves.

^5^The values given within parentheses are either the salt bridges amongst the charged+polar interactions or the aromatic-aromatic interactions amongst the hydrophobic interactions.

^6^The rest of the interactions in each category (adding up to 100%) involve charged/polar versus hydrophobic/aromatic interactions.

### Correlation with experiments

#### Correlation of interface clusters and hubs with dissociation constants

We have considered eight protein-protein complexes with known dissociation constants [50-52] and analyzed their interface cluster and hub parameters so as to correlate our results with experimentally available results on interface strength. These complexes have mainly been taken from reference [50] where a similar analysis of comparison of generic interface parameters with dissociation constants was carried out. Table 3 summarizes the results of the present interface strength analysis. The number of interface hubs (cutoff relaxed to nodes with ≥ 4 edges so as to obtain statistically significant number for analysis), the size of the largest interface cluster, the number of interface clusters (N_ic_) and the total number of residues in these interface clusters (N_ires_) at different I_min_s along with the experimentally determined dissociation constants (K_d_) are given in the table for the chosen complexes. The complexes with μM K_d _are weaker complexes and the ones with nM K_d _are the stronger ones. In general, we find that the number of interface hubs, number of interface clusters, number of interface cluster residues and largest interface cluster size are all higher for the nM K_d _complexes than the μM K_d _complexes at all I_min_s. This indicates that the interface clusters and hubs identified and the I_min _values used in the present method are genuine and robust and are good indicators of the strengths of oligomeric protein interfaces.

**Table 3:** Comparison of interface clusters and hubs with experimental dissociation constants of oligomeric proteins

Protein [Reference]	PDB Code	K_d_^1^	No. of interface hubs			Size of the largest interface cluster			N_ic _(N_ires_)^2^		
			
			I_min _(%)			I_min _(%)			I_min _(%)		
			
			0	2	4	4	6	8	4	6	8
E-Cadherin [50]	1edh	170 μM	14	3	1	41	3	0	2(44)	3(9)	0
β-Lactoglobulin [50]	1beb	20 μM	13	3	2	32	4	0	3(43)	2(7)	0
Insulin [50]	1trz	1 μM	13	8	2	31	6	0	3(37)	4(18)	0
Elongation Factor EF-TU/EF-TS Complex [50]	1efu	30 nM	54	27	11	61	18	11	8(190)	7(63)	5(28)
Rac-ExoS GAP domain [50]	1he1	20–30 nM	24	13	2	94	9	7	4(146)	4(23)	2(10)
CD4-GP120 [51]	1gc1	5 nM	28	13	5	86	15	8	4(152)	3(32)	3(21)
Ran-Importin-β [50]	1ibr	0.6 nM	49	18	7	131	15	11	6(173)	5(48)	4(22)
Growth hormone-Receptor [52]	2hhr	0.15 nM	46	21	12	257	34	22	6(344)	7(86)	5(57)

^1^: K_d_: Experimental dissociation constant

^2^: N_ic _(N_ires_): No. of interface clusters (No. of residues in the interface clusters)

#### Correlation of interface hubs with ΔΔG

Experimental results are available on the stability of interface mutants for some protein complexes [27]. These have been comprehensively presented in the Alanine Scanning Energetics Data Base, the ASEDB [53,60]. Here, we have compared our results with those from the ASEDB. We have selected those complexes from ASEDB where the dimeric structures are available, since the availability of the dimeric structure is a prerequisite for our present analysis. There are 15 such complexes in ASEDB as listed in Table 4. We then obtain the interface hubs in these complexes at different I_min_s and compare with the ΔΔG (differences in the free energies of the mutant and the wild-type) of the specific mutants given in ASEDB. We have relaxed the hub detection criterion to nodes with greater than or equal to 4 edges similar to the previous section so as to obtain statistically significant results for comparison with the experimental results. Interface hubs are identified at I_min _values of 0%, 2%, and 4% and have been characterized by the highest I_min _value at which they appear as interface hubs (since, if a residue is a hub at a particular I_min _value, then by default, it would remain a hub at all values lower than that I_min _value). Since there are very few interface hubs at I_min _> 4%, we have not considered these hubs separately in this analysis.

**Table 4:** Hot spot predictions^§ ^from interface hub analysis on protein complexes at I_min _= 4%

**PDB**	**Monomer 1**	**Predicted mutations in monomer 1**	**Monomer 2**	**Predicted mutations in monomer 2**
1A4Y	Angiogenin^1^	35L, 40K, 95R, 117Q	RNase I^1^	35D, 63R, 346Q, 349N
1CBW^2^	BPTI	17R, 37G	Chymotrypsin	57H
2PTC	BPTI	**^4^	Trypsin	57H, 99L, 190S
1BRS	Barnase	56F, 103Y	Barstar	33N
1GC1	CD4	**	GP120	280N, 368D, 370E, 469R
1DVF	D1.3	35Nh^5^, 45Lh, 50Mh, 103Lh, 106Wh, 36Yl^5^, 89Ql, 91Fl, 96Rl	E5.2	47Wh, 99Yh, 103Wh, 36Yl, 92Nl
1VFB	D1.3	35Nh, 45Lh, 40Mh, 103Lh, 106Wh, 36Yl, 44Pl, 89Ql, 91Fl, 96Rl	HEL	27N
1DAN^3^	Factor VII	128Fh, 164Mh, 208Yh, 230Rh, 95Nl, 101Yl, 118Yl	Tissue Factor	19F, 74R, 96N, 100F, 147F
3HFM	HEL	**	HYHEL-10	33Yh, 39Kh, 50Yh, 98Wh, 103Wh, 166Fh, 32Nl, 50Yl, 94Wl, 96Yl, 121Sl, 123El, 135Fl
3HHR^3^	HGH	8R, 9L, 12N, 16R, 41K	hGHBP	150H, 152D, 197V, 200Y, 217R, 218N
1BXI	IM9	**	E9DNase	72N, 75N, 84S, 86F, 97K
1FC2	Protein A	**	IgG1	30M, 31I
1DFJ	RNaseI	**	RNase A	41K, 111E
1JCK	SEC3	**	TcrVb	43L, 101Y, 108F
1AHW	Tissue Factor	152I, 169K, 171N, 190Q, 192V, 201K	Fab 5G9	32Yl, 36Yl, 50Yl, 91Hl, 135Fl, 137Nl, 33Yh, 35Hh, 45Lh, 50Lh, 52Dh, 59Ih, 102Yh, 103Yh, 104Fh, 147Kh, 170Fh

^§ ^Already mutated hubs have been excluded from this table.

^1 ^The underlined monomers are the ones where some mutations have already been carried out.

^2 ^No hubs are obtained at I_min _= 4% in 1CBW. Hence, hubs at I_min _= 2% are used for prediction.

^3 ^1DAN and 3HHR are proteins where a large number of mutations (>100) have been carried out, which have ΔΔG < 1 kcal/mol. These mutations have not been included in this analysis.

^4 ^** All hubs identified at I_min _= 4% have already been mutated.

^5 ^h or l accompanying a residue name corresponds to the heavy and the light chains of the corresponding antibodies.

**Figure 5:**
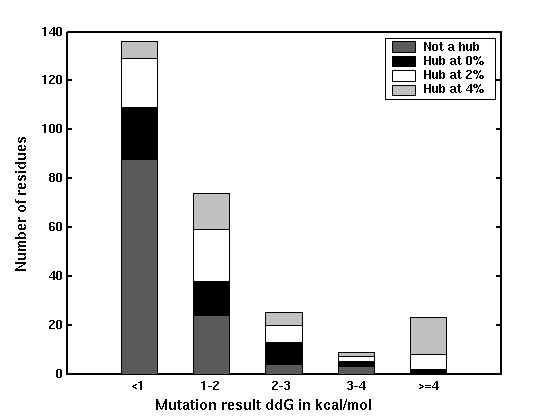
Correlation of ΔΔG with hubs obtained at different I_min_s. The experimental mutations are categorized into different bins based on their ΔΔG values in kcal/mol. The distribution of the hub character of the residues (highest I_min _at which a residue is a hub) is shown in different shades within these bins.

Figure 5 summarizes the overall results of this analysis pertaining to the 15 complexes. The mutation results are categorized as those with ΔΔG in the ranges of <1, 1–2, 2–3, 3–4 and ≥ 4 kcal/mol. The frequency distribution of the mutated residues, according to their hub character is presented for different ΔΔG values in Figure 5. We find that a majority of the mutations with ΔΔG < 1 kcal/mol are not hubs at any I_min _whereas, most of the mutations with ΔΔG ≥ 4 kcal/mol are interface hubs at I_min _= 4% (though there are some hubs at I_min _= 2% and I_min _= 0%). The fraction of hubs at I_min _= 4% with ΔΔG < 1 kcal/mol is insignificant and there is no mutation with ΔΔG ≥ 4 kcal/mol, which is not a hub. Mutations in the 1–2, 2–3 and 3–4 kcal/mol ranges, do show a combination of hub characters, which are however consistent over the range. In general, we find that the interface hubs obtained at higher I_min _values (I_min _= 4%) have higher ΔΔG values than the hubs obtained at lower I_min _values (I_min _= 0%) and the residues that do not form hubs at all. Hence, the interface hubs identified using the present method correlate well with the experimentally obtained ΔΔG values of the interface hot spots.

Out of the already mutated residues, ten are found to be hubs even at I_min _= 6%, out of which, five have ΔΔG ≥ 4 kcal/mol and the other five have ΔΔG varying between 1 and 4 kcal/mol. None of these have ΔΔG < 1 kcal/mol. One of the residues in the Trypsin-BPTI complex (Lysine 15) is known to have a ΔΔG ≈ 10 kcal/mol [54] and this residue remains a hub even at I_min _= 8%. This happens to be the only residue with such a high ΔΔG value and also the only one to remain a hub even at I_min _= 8%.

Surprisingly, a large number of the interface hubs identified by the present method in these complexes, have not been mutated (not shown in figure). These include quite a few strong hubs identified at I_min _= 4% (84 in number). These have been listed in Table 4 and are potential hot spots in these protein complexes, which can be mutated to destabilize the protein interface. It would be interesting to verify these predictions experimentally, which would then establish this as a rational method for the design of mutants that disrupt the protein-protein interfaces.

## Conclusion

The oligomeric protein structures have been represented as networks, with amino acid residues as nodes and the edges have been constructed on the basis of non-covalent interaction strength (ranging from a cutoff of 0% to 6%) between amino acids. The analysis is focused on characterizing the interface clusters and hubs.

The interfaces have been characterized as strong, if the largest cluster in the protein appears at the interface at high (6%) interaction strength. Interestingly more than 50% of the complexes in the dataset exhibit such strong interfaces. The interface clusters identified and their amino acid composition correlate with those identified from previous studies as well as from δASA calculations.

The composition and the connections of the highly connected interface hubs have been evaluated at varying interaction strengths and compared with those of the non-interface hubs. The interfaces show an increase in Arginine hubs and a decrease in hydrophobic hubs when compared to the non-interface hubs. The hydrophobic residues, though present in the interface clusters, do not contribute to the interface hubs. Further, the interface hubs make the usual interactions such as salt bridges, stacking interactions and hydrogen-bonds as well as unusual interactions such as Arginine-Arginine interactions. The hub and non-hub interactions at the interfaces also show specific profiles with the hub interactions being dominated with hydrophobic interactions at lower interaction cutoffs and charged interactions at higher interaction cutoffs, whereas the non-hub interactions are dominated with charged interactions at all cutoffs. More importantly, the cluster and hub identification procedure picks up all types of interactions in a consolidated way, giving a global view of the interactions at the interface.

The interface clusters and hubs identified correlate well with the experimentally determined dissociation constants for known complexes indicating that we have a robust method of identifying the strength of oligomeric protein interfaces. Finally, the hubs at high interaction strength have been identified as hotspots by comparing the ΔΔG values from alanine scanning mutagenesis experiments. Several strong hubs that have not been mutated have been predicted to be hotspots and await confirmation from future experiments.

## Materials and methods

### Dataset

The dataset consists of a non-redundant set of protein multimer (455 in number) structures with resolution better than 2 Å, obtained from the protein data bank [55]. The dataset list is provided in Table A1 in Additional file 1. The sequence identity of the selected proteins is less than 25%. In the cases where the full multimer coordinates were not provided, they were generated from the rotation matrices and translation vectors. The dataset includes dimers and multimers of all types such as homo, hetero, functional as well as crystallographic multimers. 44 of the 455 oligomers (<10%) are crystal dimers as obtained from the BIOLOGICAL_UNIT record of the pdb file and the protein quaternary structure server [56]. These proteins are indicated in Table A1 (see Additional file 1). The size of the monomers varies from 50 to 1000 and that of the multimers varies from 100 to 2500.

### Accessible surface area

The loss of accessible surface area upon dimerization/multimerization was calculated from the residue-wise accessible surface area of the multimeric proteins and that of their respective monomers, which were obtained from NACCESS [57]. The multimer values were normalized to those of the dimers. The residues that lose greater than 1% of their accessible surface area upon dimerization were identified as those contributing to the interface from δASA calculations.

### Network construction

#### Definitions

Protein structures have been considered as a network of interactions amongst amino acid residues. Each residue in a protein complex is considered as a *node *in the graph and the connections between these nodes are the *edges*. A group of interconnected nodes is defined as a *cluster *and a cluster with at least one residue belonging to a different protein chain in the multimer is denoted as an *interface cluster*. *Contact number *is defined as the number of edges made by a node and those nodes with a contact number greater than 4 (unless otherwise specified), have been identified as *hubs*. A hub with at least one residue belonging to a different protein chain in the multimer is denoted as an *interface hub*.

### Evaluation of non-covalent interaction

The non-covalent interactions between side chain atoms of amino acid residues (with the exception of Glycine, where the C^α ^atom is taken) are considered. The interactions between the sequence neighbors however, have been ignored. The interaction between two residues i and j has been quantified as defined by Kannan and Vishveshwara [58]:

I_ij _= (n_ij_/*N*) × 100

where n_ij _is the number of atom pairs belonging to the side-chains of i and j coming within a distance of 4.5 Å and *N *is the normalization value for the amino acid type, which has been evaluated previously from a non-redundant set of proteins and also correlates with the size of the residue [58]. The lesser of the two normalization values corresponding to the residues i and j is used for the evaluation of the interaction I_ij _for *cluster *identification. The normalization value of the residue i is used to evaluate the interaction I_ij_, for *hub *detection. In the identification of the *clusters*, both the normalization values of residues i and j are required during I_ij _evaluation due to symmetric considerations during graph construction. We have tried different combinations of the normalization values in this case, like sqrt(*N*_i _× *N*_j_), (*N*_i _+ *N*_j_)/2 and min(*N*_i_, *N*_j_). Since they give qualitatively very similar results, we use the lesser of the two values (min(*N*_i_, *N*_j_)) for cluster identification. However, for *hub *detection, such constraints are not there and hence we have used the normalization value of the residue i (*N*_i_) whose *hub *character is being evaluated.

### Contact criterion on the basis of interaction strength

We choose an interaction cutoff, referred to as I_min _and any two non-sequential ij pair, which has an I_ij _value that is greater than a chosen I_min _value, is connected by an edge in the graph. Such a graph is referred to as a protein structure graph for a given interaction strength I_min_. The protein structure graphs are generated for all the multimers considered in the dataset using an I_min _range varying from 0 to 10%. Physically, a higher I_min _indicates strong interactions between the connected residues and a lower I_min _includes the weakly interacting residues as well. For instance, at I_min _= 0% even a single atom-atom contact between the side-chains of two residues is sufficient to connect them by an edge in the protein structure graph and more contacts are required for connections at higher I_min_s. The interface clusters and hubs were identified and analyzed in these protein structure graphs at varying I_min_s. Finally, an I_min _of 6% was chosen for interface cluster analyses due to better correlation with results from δASA and an I_min _of 0% to 4% was chosen for interface hub analyses so as to obtain statistically significant number for analyses.

### Cluster and hub analysis

The protein structure graphs have been represented as an adjacency matrix, which is an N × N matrix, where N is the number of residues in the protein structure. Each ij^th ^element in the matrix is either 0 or 1 depending on whether the two nodes (residues) are connected (interacting) or not, on the basis of the chosen I_min_. The diagonal elements are considered as 0 since connections with self are avoided. The amino acid residues forming disjoint *clusters *(with minimum three residues in each) are identified from the adjacency matrix by using a standard graph algorithm (depth first search (DFS) algorithm [59]). This gives the *clusters *of all the interacting residues in the protein structure, from which the *interface clusters *are selected.

Similarly, the residues with *contact number *greater than 4 are detected as *hubs*, from which the *interface hubs *are identified. The *hub *definition is relaxed to a *contact number *equal to or greater than 4, while investigating the *interface hubs *of single multimeric complexes in detail, as given in Tables 3 and 4, in order to obtain statistically significant number for analysis. The *interfacial hub *preferences of amino acid residues and the preferences of the residues with which these *hubs *interact are obtained and compared with similar properties of the *non-interface hubs *and *non-hubs *at interfaces, identified from the same data set.

### Size of the largest cluster

When analyzing complex networks, one of the most common parameters used is the size of the largest cluster [42]. Here, we have used this parameter to analyze the structure networks of protein oligomers. At various I_min_s, the clusters in the protein oligomers are obtained using DFS and the size of the largest cluster in terms of the number of residues constituting it is obtained at different I_min_s. This has been found to be a function of protein size and hence the size of the largest cluster is normalized with respect to the protein size and is plotted as a function of I_min_. The largest cluster size decreases as the I_min _increases and the largest cluster obtained at a higher I_min _may or may not be present at the oligomeric interface. An analysis is made on all the proteins in the data set, to find out if the largest cluster is at the interface or not at I_min _= 6%. This provides an idea regarding the strength of the oligomeric interface with respect to its monomeric protein core.

## Authors' contributions

KVB carried out the construction and analysis of oligomeric protein structure networks. SV devised the concepts and the formalism used in this study. Both authors contributed to the interpretation of results and the preparation of the manuscript.

## Supplementary Material

Additional File 1 Two tables (Table A1 and Table A2) are provided as additional material (see Additional file 1), giving the list of pdbs in the dataset and the 20 × 20 matrix for the residue preferences of the non-interface hubs to interact with the 20 different amino acid types at I_min _= 4%, respectively. Two figures (Figure A1 and Figure A2) are also provided as additional material in Additional file 1, giving the correlation of δASA with interface clusters and the amino acid composition in the interface clusters, respectively. All four additional materials (two tables and two figures) are provided as a single word document (Additional file 1). Click here for file
