# Supplementary material for: Oligomeric protein structure networks: insights into protein-protein interactions
Source: BMC Bioinformatics. 2005 Dec 10;6:296. doi: 10.1186/1471-2105-6-296 (PMC1326230; doi:10.1186/1471-2105-6-296)
Supplement: Additional File 1 — Two tables (Table A1 and Table A2) are provided as additional material (see Additional file 1), giving the list of pdbs in the dataset and the 20 × 20 matrix for the residue preferences of the non-interface hubs to interact with the 20 different amino acid types at Imin = 4%, respectively. Two figures (Figure A1 and Figure A2) are also provided as additional material in Additional file 1, giving the correlation of δASA with interface clusters and the amino acid composition in the interface clusters, respectively. All four additional materials (two tables and two figures) are provided as a single word document (Additional file 1). [file 1471-2105-6-296-S1.pdf]

Additional Material

Table A1: Dataset of oligomers used in the analysis

**Table A1a: List of protein complexes with interface stronger than the monomeric protein core [1,2,3,4]**
1a68 1a8i 1al3 1aw8 1az9 1b2p 1b4k 1b5q 1b78 1b8f 1bgf 1bgv 1bif 1byr 1c3c
1c3m 1c4q 1c5e 1cxp 1cxq ***1czp*** 1d02 1d0i 1d1q 1d3y 1d7c ***1d7y*** 1d8d 1dbf 1dc1
1df4 1dfm 1dfo 1dg6 1dgw 1dhp 1dhs 1di6 ***1dk8*** 1dmh 1dmu 1do6 1dqa 1dtd 1duv
1dv1 1dwk 1dxe 1dxp 1dxy 1dyo ***1e30*** 1e3d 1e4c 1e4m ***1e58*** ***1e5p*** 1e6b 1e85 1e9e
1eaj 1ec7 1ecs 1ed8 1eex 1ef8 1eg9 1ei7 1ejb 1elq 1enp 1ep0 1epx 1erz 1euv
1euw 1evx ***1ew6*** 1eyr 1eyv 1eyz 1ezw 1f06 1f1b 1f2t 1f2v 1f61 1f74 1f7l 1f86
1f8y 1fa8 ***1fc3*** 1fe6 1fiu 1fjs 1flm 1fm0 1fns 1fq0 1fr3 1fs7 1fsg 1fws ***1fx2***
1fxo 1fzr 1g0s 1g2i 1g2q 1g57 ***1g61*** 1g6u 1g72 1g8m 1g8q 1g99 ***1gcp*** 1gd0 1gde
1ge7 1gm7 1gsa 1h54 ***1h6g*** 1h72 1h7e 1hbz 1he1 1hnj 1huv 1hw1 1hx6 1hy0 1hyo
1hz6 1i0h 1i0s 1i2o 1i4f 1i4u 1i6p 1i86 1i8d 1iat 1iby 1icf 1ih8 1ihr 1inl
1ird ***1j83*** 1j98 1j9q 1jb0 1jd0 1jh8 1jhg 1jiw 1jjy 1jm6 1jnw 1jq5 1jqc 1js3
1jsh 1jtg 1jw9 1jxz 1jy3 1jzt 1k9s 1mof 1nox 1pdo 1pjc 1pym 1qcz 1qf5 1qfx
1qh4 1qh8 1qhd 1qhv 1qi9 1qj2 1qj5 1qlm 1qlw 1qmv ***1qmy*** 1qna ***1qo2*** ***1qps*** 1qqf
1qre 1qrr 1qsm 1qtn 1qto 1qu9 1rh4 1rux 1scf 1svb 1svp 1swu 1taf 1thf 1tyv
1vie 1vls 2pii 3tdt 4pga 4tsv 4ubp 5csm 7odc 9gaf

**Table A1b: List of protein complexes with interface weaker than the monomeric protein core [1,2,3,4]**
1b0u 1b16 1b6r 1b71 1b8g 1bg6 1c0p 1c3w ***1c9o*** 1ccw 1d1v 1d4x 1d8w 1dbw
1dii 1dj0 ***1dk0*** 1dku 1dkz 1dmm ***1dp4*** ***1dqz*** 1dvj ***1dw0*** ***1dy5*** ***1dys*** 1e44 1e54
1e5m 1e6u 1eb7 1ei1 1ejd 1ek6 1ep3 1es9 1esg 1eud 1eum 1evy 1ewk 1ex2
1eye ***1f0l*** 1f44 1f46 ***1f4l*** 1f60 1f6d 1fds 1ffg 1fi2 1fj2 1fjh 1fjj 1fn9
1fp3 1fp6 ***1fqt*** 1fsi 1fx8 1fyh ***1g2a*** 1g3k 1gew 1ghq 1gl1 ***1h4x*** ***1h6r*** 1h9s
1hdh ***1hf8*** 1hoz 1hxx 1hzy ***1i19*** 1i45 ***1i7k*** 1i7q ***1iaz*** 1im8 1j79 ***1j97*** 1j9l
1jcl 1jhd 1jil 1jnp 1jp3 ***1jpz*** 1jr2 1jr7 1k20 1nkd 1qd1 1qf8 1qfe 1qft
1qj4 ***1qjv*** 1ql0 ***1qme*** 1uox 1vqb 2dtr ***2thi*** 4bcl ***4pah***

[1] **Table A1a and A1b put together constitute the whole dataset.**

[2] **Interface strength calculated according to the presence/absence of largest cluster at the interface at I_{min}=6% (as explained in the text).**

[3] **Some of the complexes have different oligomeric interfaces constituting them. When those are included, the number increases to 455 from the 328 given here.**

[4] **The crystal oligomers (as identified from the protein quaternary structure server [56] and "BIOLOGICAL_UNIT" data from the pdb file) are indicated in bold and *italics*. These fall mainly, but not necessarily, in the weak oligomers list (Table A1b). It is to be noted that the strong and weak categorization of the oligomeric protein interfaces carried out here, is with respect to the corresponding monomeric protein cores and not with respect to each other.**

Table A2: Preferences of non-interface hubs to interact with other residues at I_{min}=4%[1]

Res	H_n	Ala	Arg	Asn	Asp	Cys	Gln	Glu	Gly	His	Ile	Leu	Lys	Met	Phe	Pro	Ser	Thr	Trp	Tyr	Val
ALA	0.00	0.00	0.00	0.00	0.00	0.00	0.00	0.00	0.00	0.00	0.00	0.00	0.00	0.00	0.00	0.00	0.00	0.00	0.00	0.00	0.00
ARG	3.15	1.82	4.95	4.50	16.07	0.89	5.47	12.42	0.56	4.28	3.83	7.25	1.56	2.64	5.43	1.71	4.20	4.87	3.98	9.56	4.02
ASN	1.82	1.76	4.04	8.51	8.29	0.51	3.89	6.24	2.49	4.11	5.94	6.60	2.57	2.35	6.46	4.18	7.48	9.61	3.96	7.19	3.82
ASP	0.37	0.28	12.61	4.76	6.44	0.00	3.08	4.76	0.00	12.89	5.60	4.48	2.80	1.68	7.28	1.68	5.32	7.84	5.60	10.08	2.80
CYS	0.14	0.00	0.00	0.00	16.00	0.00	16.00	28.00	0.00	4.00	0.00	0.00	20.00	0.00	0.00	0.00	16.00	0.00	0.00	0.00	0.00
GLN	1.17	3.50	5.79	4.98	7.13	1.48	5.52	10.50	0.67	4.58	3.36	3.50	3.90	2.56	11.98	2.02	5.65	6.46	4.17	7.67	4.58
GLU	1.33	0.66	9.82	5.80	3.23	1.52	5.01	4.55	0.72	9.03	5.67	5.93	7.77	2.90	8.37	3.49	8.10	4.41	1.52	5.47	6.06
GLY	0.00	0.00	0.00	0.00	0.00	0.00	0.00	0.00	0.00	0.00	0.00	0.00	0.00	0.00	0.00	0.00	0.00	0.00	0.00	0.00	0.00
HIS	4.52	3.04	5.14	3.98	9.78	1.49	2.82	10.00	0.33	11.71	3.92	8.51	1.82	2.65	6.96	1.66	3.76	4.92	3.26	8.56	5.69
ILE	1.09	1.24	3.71	4.86	2.48	0.38	4.19	4.48	0.38	2.76	6.29	17.05	1.81	3.05	14.76	3.52	3.05	2.19	3.05	10.10	10.67
LEU	1.44	1.57	3.99	4.08	2.55	0.72	3.41	4.03	1.12	2.55	9.10	16.41	1.57	4.80	12.46	1.88	2.96	5.60	3.99	9.50	7.71
LYS	1.81	0.49	3.40	2.07	10.33	0.36	4.80	14.29	0.24	2.25	3.34	5.23	3.04	5.78	6.14	2.61	3.71	7.66	3.22	14.77	6.26
MET	3.54	0.75	3.82	1.87	3.22	1.80	3.37	2.32	0.67	2.85	9.15	13.72	1.57	6.30	14.47	2.17	1.27	3.45	4.87	10.49	11.84
PHE	8.09	1.92	3.53	3.89	1.94	1.10	3.32	3.44	1.40	2.61	9.53	12.16	2.45	3.98	15.75	3.04	3.64	3.18	6.11	8.65	8.36
PRO	0.17	0.00	6.15	0.00	3.85	0.00	2.31	1.54	0.00	4.62	3.85	6.92	1.54	4.62	8.46	3.08	3.08	3.08	10.77	15.38	20.77
SER	0.02	0.00	0.00	12.50	18.75	0.00	6.25	0.00	0.00	6.25	0.00	6.25	0.00	0.00	12.50	6.25	0.00	25.00	0.00	6.25	0.00
THR	0.38	0.82	3.28	9.84	13.39	0.82	3.28	7.38	0.55	6.56	4.10	4.10	4.64	1.37	11.20	4.10	4.64	4.92	1.64	7.92	5.46
TRP	11.61	1.99	4.83	4.36	5.96	0.90	4.64	3.74	0.74	2.26	7.40	9.78	3.16	3.35	12.70	4.64	3.43	3.82	5.57	10.72	6.00
TYR	4.95	1.32	6.25	3.67	6.35	0.71	3.43	5.36	1.53	2.34	7.81	7.20	5.53	4.34	11.41	5.43	3.16	2.92	5.40	9.95	5.91
VAL	0.34	0.45	3.85	2.95	5.90	0.45	1.36	5.90	0.45	4.76	7.94	11.11	6.80	3.40	11.79	6.12	1.13	4.31	4.08	11.79	5.44

[1]: The rows of the table (i) correspond to the twenty different types of amino acids forming the non-interface hubs. The second element in each row gives H_n, the percentage of amino acids of each type 'i' forming non-interface hubs in the dataset (with respect to the total number of residues of type 'i' in the dataset). Elements three to twenty two in each row correspond to the percentage of interactions that the amino acid hub of type 'i' makes with the twenty different amino acid types (j) [with respect to the total number of interactions made by hub residue type 'i']. This gives the 20×20 matrix for the interactions of the non-interface hubs with other residues at I_{min}=4%. Each ij^{th} element in the 20×20 matrix gives the percentage interactions of non-interface hub type 'i' with residue type 'j'.

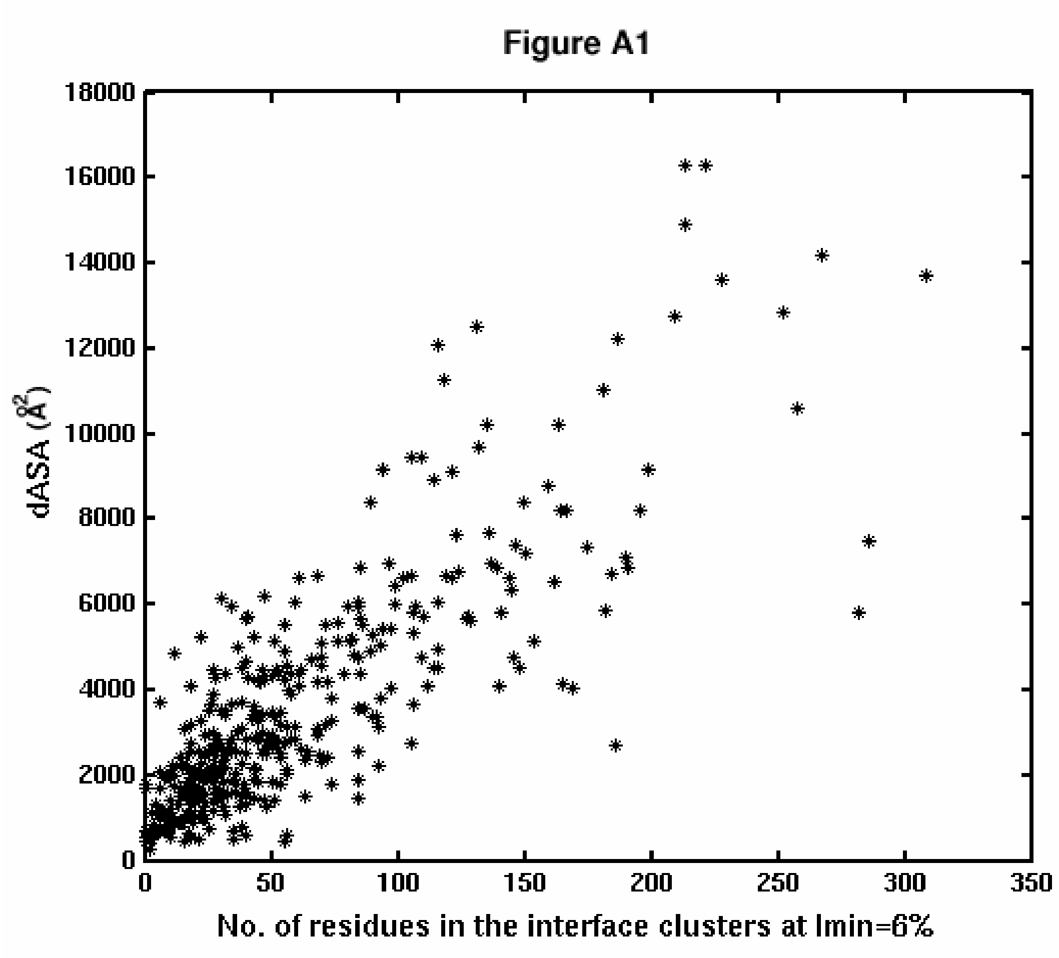

Figure A1: Plot of the accessible surface area lost on oligomerization (δASA in Å^2) Vs number of residues in the interface clusters as obtained at I_{min}=6%. Both the values have been normalized to that of the dimers in case of the multimers. This figure illustrates the point that the residues in the interface clusters obtained at I_{min}=6% is well correlated with δASA (correlation co-efficient = 0.83). Such a correlation is also seen at I_{min}=4% (correlation co-efficient = 0.70, not shown in figure), but is better at I_{min}=6%. However, at lower I_{min} values, for example at I_{min}=0%, this correlation decreases further (correlation co-efficient = 0.45), since the interface clusters include many more residues from the monomeric protein core, which do not necessarily contribute to the interface. Similarly, at I_{min} values beyond 6%, we get tightly interacting small clusters at the interface, which fail to represent the complete interface. The higher I_{min}s (above 6%) can be used to identify residues that strongly interact across the interface and to characterize the interface of specific protein complexes. However, in the present analysis, where a large dataset consisting of different interface types is present, I_{min}=6%, which correlates very well with δASA, has been chosen for identifying interface clusters. This I_{min} value offers a good trade off between specificity and selectivity.

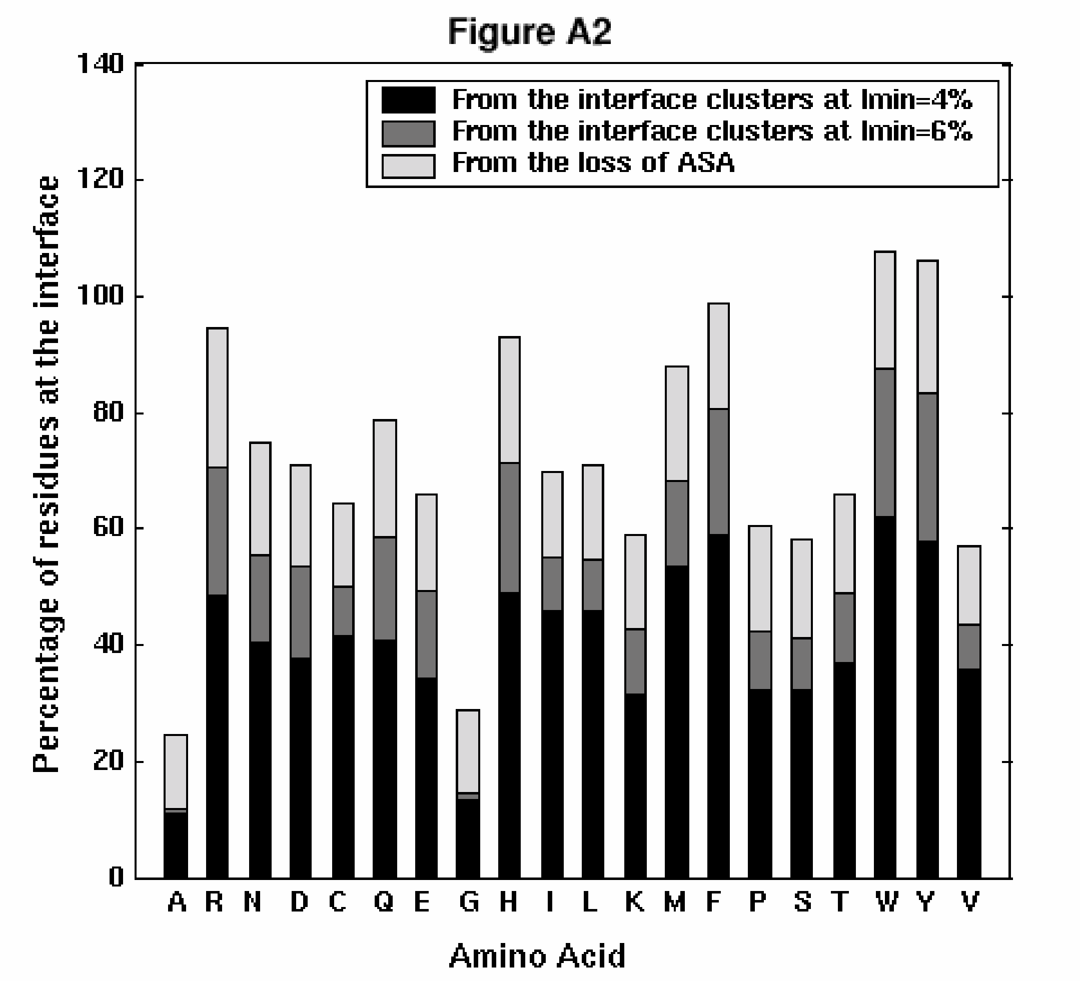

Figure A2: Amino acid preferences in the interface clusters at I_{min}=4%, I_{min}=6% and as obtained from δASA calculations. The percentage compositions of amino acid residues in the interface clusters and from δASA are presented (i.e., (No. of residues of type 'i' in the interface clusters or from δASA ÷ Total No. of residues of type 'i' present in the dataset) × 100). The residue compositions obtained from the interface clusters at I_{min}=6% correlate well with those obtained using the δASA calculations for all residues except in the case of small amino acids like Glycine and Alanine, for which the correlation is better at I_{min}=4%. Almost all the amino acid types make significant contribution to the interface clusters. However there seems to be a preference for Arginine, Histidine, Tryptophan, Tyrosine and Phenyl alanine at I_{min}=6%.
